# Mesenchymal stromal cells in the thymus

**DOI:** 10.1186/s41232-022-00219-5

**Published:** 2022-11-02

**Authors:** Takeshi Nitta

**Affiliations:** Department of Immunology, Graduate School of Medicine and Faculty of Medicine, The University of Tokyo, Tokyo, Japan

**Keywords:** Thymus, Mesenchymal cell, Fibroblast

## Abstract

The microenvironment of the thymus is composed of a group of stromal cells that include endoderm-derived thymic epithelial cells (TECs) and mesenchymal stromal cells such as fibroblasts and serves as a site for the development of T cells. TECs are known to play an essential role in T cell differentiation and selection. Mesenchymal stromal cells have been less studied in terms of their immunological significance compared to TECs. Recently, new technologies have made it possible to identify and characterize mesenchymal stromal cells in the thymus, revealing their unique functions in thymic organogenesis and T cell development. This review outlines the current views on mesenchymal stromal cells in the thymus, particularly highlighting the newly discovered function of thymic fibroblasts in T cell repertoire selection.

## Background

**Fig. 1:**
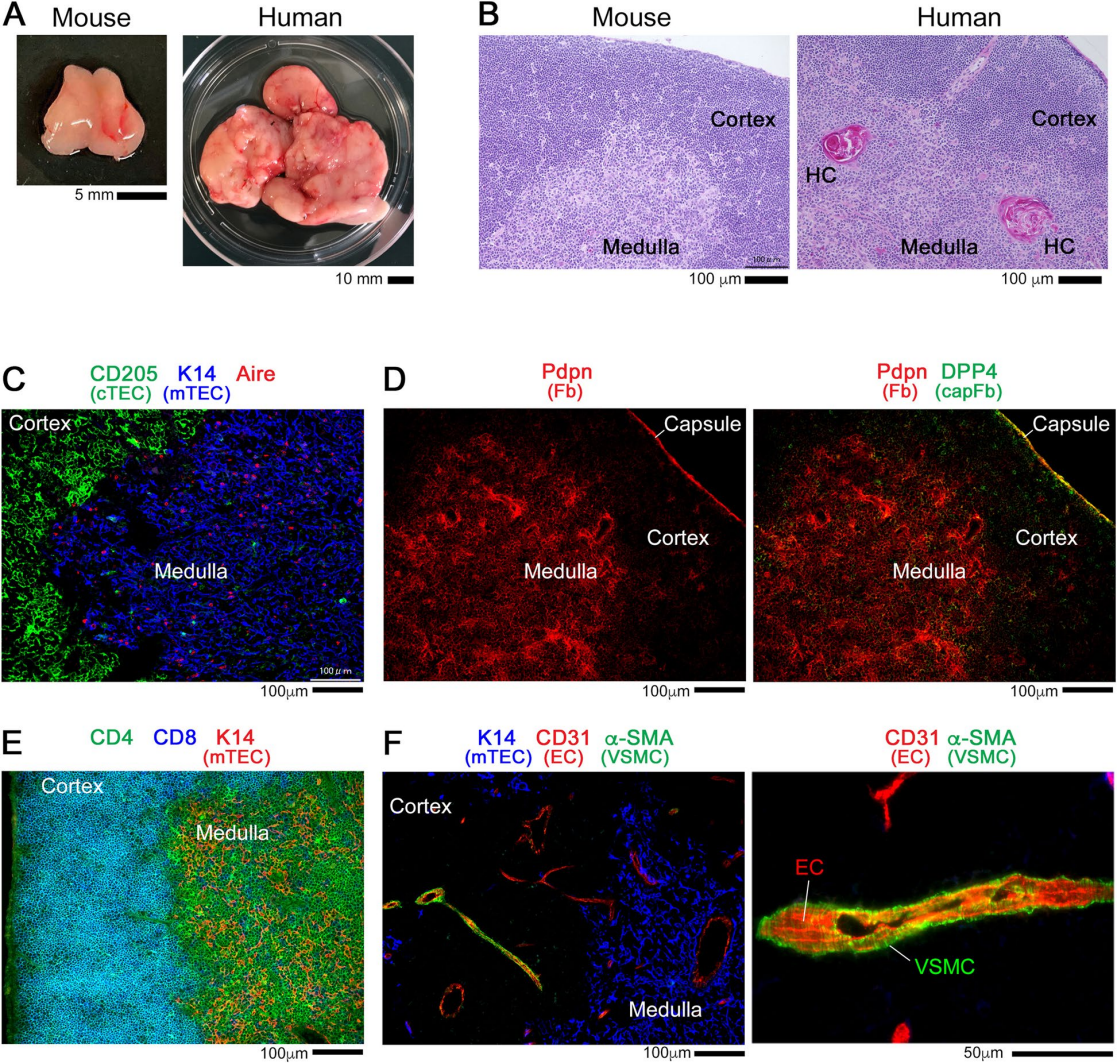
Histological detection of thymic stromal cells. **A** Photographs of thymus from wild-type C57BL/6 mouse (5-week-old) or human (7-month-old). **B** Hematoxylin and eosin staining of thymus sections from C57BL/6 mouse (5-week-old) or human (7-month-old). In the human thymus, Hassall’s corpuscles (HCs), one of the final differentiation state of mTECs, are prominently detected within the medulla. **C**–**F** Thymus sections from 5-week-old C57BL/6 mice were stained for the indicated markers: CD205 (cTEC), keratin 14 (K14) (mTEC), Aire (mTEC), Pdpn (fibroblast, Fb), DPP4 (capFb), CD4, CD8, CD31 (endothelial cell, EC), and α-SMA (VSMC)

The thymus is an organ with a white appearance (Fig. 1A). The reason for this is that the thymus is densely filled with white blood cells, mainly immature T cells called thymocytes that greatly outnumber red blood cells and blood vessels. Thymocytes are derived from early T cell progenitors (ETPs) from the fetal liver or bone marrow, mature into T cells and acquire the antigen recognition repertoire of the T cell receptor (TCR) in the thymus, and exit into the peripheral circulation [1]. In other words, the thymus is a dynamic organ in which thymocytes, which comprise the majority of its volume, are constantly being replaced. The framework of this thymocyte-hosting organ consists of a three-dimensional meshwork structure composed of various stromal cells [2–4]. In this review, the nature and function of these thymic stromal cells will be discussed.

In mammals, the thymus is a two-lobed organ located above the heart. The surface of the thymus is covered with a capsule composed of fibroblasts. The microenvironment within the thymus parenchyma can be subdivided into two anatomically discrete regions, the cortex and medulla, wherein different sets of developing thymocytes and supporting stromal cells reside. The cortex is the outer region where immature thymocytes are densely packed within the stromal architecture, while the medulla is the inner region, where mature thymocytes are supported by a meshwork of stromal cells at a lower density (Fig. 1B). These two regions in the thymus are characterized by distinct subsets of thymic epithelial cells (TECs), cortical TECs (cTECs), and medullary TECs (mTECs) (Fig. 1C), which provide the meshwork architecture of each region and play an essential role in generating functional T cells [5, 6].

TECs are endoderm-derived and are the most prominent thymic stromal cells. In addition to TECs, a variety of other stromal cells of different origins support the thymic microenvironment [7, 8]. Mesenchymal cells are representative non-TEC stromal cells in the thymus and are predominantly found as fibroblasts in the capsule (capsular fibroblasts, capFbs) and medulla (medullary fibroblasts, mFbs) in the postnatal thymus [4] (Fig. 1D). During embryogenesis, the mesenchymal cells are important for the organogenesis of the thymus and differentiation of TECs. The blood vasculature is also an important component of the thymus parenchyma [9]. The cortex has a network of capillaries, and the medulla contains arterioles and postcapillary venules. In particular, the corticomedullary junction (CMJ) region is enriched with blood vessels that provide the entry site for ETPs and exit site for mature T cells.

This review will start with a description of how T cells develop and acquire their TCR repertoire in the thymic microenvironment, which is composed of such diverse stromal cells, first focusing on the function of TECs.

### Thymic epithelial cells (TECs)

Following the seeding of the thymus through the blood vessels at the CMJ, ETPs differentiate into CD4^-^CD8^-^ (double negative, DN) thymocytes in the thymic cortex [10]. Upon interaction with cTECs, DN thymocytes are induced to commit to the T cell-lineage and undergo rearrangement of the genes encoding the T cell receptor (TCR) [11, 12]. DN thymocytes proliferate as they migrate from the CMJ outward through the cortex, turning back at the outermost region (called subcapsular zone) to differentiate into CD4^+^CD8^+^ (double positive, DP) thymocytes (Fig. 1E).

DP thymocytes that have completed a rearrangement of the TCR genes in the cortex express the TCR on their surface and undergo selection according to their interaction with self-peptide/MHC (pMHC) complexes presented in cTECs [13]. DP thymocytes with a functional TCR that moderately interact with self-pMHC are induced to differentiate into CD4^+^CD8^-^ (CD4 single positive, CD4SP) or CD4^-^CD8^+^ (CD8SP) thymocytes (positive selection), while cells with no functional TCR and cells expressing TCR that strongly interact with self-pMHC are deleted by apoptosis (null selection and negative selection, respectively). cTECs have unique proteolytic machinery with a high expression of proteasome catalytic subunit and lysosomal proteases, which produce a unique set of self-peptides that enables efficient positive selection [14–18].

Recent studies have suggested that certain individual cTECs serve as a giant cell that interacts with hundreds of DP thymocytes [19, 20]. In particular, a fraction of cTECs forms large multicellular complexes, termed “thymic nurse cells,” that enclose multiple DP thymocytes alive within intracellular vesicles [21]. These unique cell-in-cell structures facilitate prolonged survival and continued rearrangement of the TCRα chain in enclosed DP thymocytes, contributing to a maximizing of the diversity of the TCR repertoire. The functional diversity of cTECs is still largely unknown, despite recent advances in single-cell transcriptome analysis [6]. The cortex also contains small numbers of dendritic cells (DCs) that contribute to negative selection.

Positively selected SP thymocytes migrate to the medulla (Fig. 1E), where they undergo further selection processes, deletion of autoreactive cells, and the induction of differentiation into regulatory T cells (Tregs). mTECs express a large number of genes that correspond to most of the coding transcripts, including those of the peripheral tissue-restricted antigens (TRAs) [22, 23]. Expression of these genes in mTECs is achieved by the nuclear protein Aire and other transcription factors [6, 24–26]. A number of gene products, including the TRAs expressed in mTECs, are presented as self-antigens directly by mTECs or indirectly by DCs to induce negative selection and/or Treg conversion of SP thymocytes that strongly interact with self-antigens. mTECs contain several functionally distinct subsets, one of which produces the chemokine CCL21 that induces the relocation of SP thymocytes from the cortex to the medulla [27–30]. A fraction of mature mTECs undergoes terminal differentiation into atypical epithelial cells such as corneocyte-like cells forming Hassall’s corpuscles and thymic tuft cells, likely contributing to the diversification of peripheral antigen expression [31–37]. In addition to mTECs, the thymic medulla contains stromal networks of mFbs and blood vessels composed of endothelial cells and mural cells, each of which has a unique role in maintaining the medullary microenvironment and controlling T cell development.

Through these stepwise differentiation processes from the thymic cortex to the medulla, mature T cells with a diverse yet self-tolerant TCR repertoire are generated and migrate to the periphery to exert immune responses.

### Mesenchymal stromal cells in thymic organogenesis

In order to understand the positioning and interrelationship of thymic stromal cells, it is necessary to look back at the process of thymic organogenesis. The thymus, along with the parathyroid glands, arises from the 3rd pharyngeal pouch (PP), which is an embryonic structure formed by the endoderm-derived epithelial layer evaginating from the foregut towards both sides [38]. These epithelial cells are the origin of both cTECs and mTECs in the adult thymus. As the 3rd PP forms, the evaginating epithelial cells are surrounded by mesenchymal cells derived from the neural crest (NC) cells in this region. With the support of these mesenchymal cells, the epithelial cells proliferate and differentiate, with cells in the caudal ventral region giving rise to the thymus and those in the cranial dorsal region forming the parathyroid glands. While the epithelial cells are differentiating into TECs and organizing the thymus parenchyma, a fraction of the surrounding mesenchymal cells and mesoderm-derived endothelial progenitor cells migrate into the parenchyma across the epithelial layers to establish an intrathymic fibroblastic network and vasculature [39, 40]. Another set of mesenchymal cells stay outside the thymic parenchyma and form a capsule. Thus, the spatiotemporal coordination by endoderm-derived epithelial cells, NC-derived mesenchymal cells, and mesoderm-derived endothelial cells is crucial for the thymic organogenesis. T cell progenitors enter the thymic parenchyma prior to the mesenchymal and endothelial cells and initiate the process of T cell differentiation.

The transcription factor FoxN1, expressed in the epithelial cells in the thymus primordium, is a master regulator of TEC differentiation during thymic organogenesis and is also essential for the function of TECs in the adult thymus [41]. Other transcription factors expressed in mesenchymal cells in this region, including HoxA1, Eya1, Six1, Pax9, Tbx1, and Ripply3, are also required for the patterning of the 3rd PP and the subsequent development of the thymus [42]. Genetic defects in these transcription factors result in thymus hypoplasia and severe immunodeficiency in humans, indicating the importance of mesenchymal cells in thymic organogenesis.

Early studies with reaggregated organ culture of isolated fetal thymic stromal cells showed that not only TECs but also mesenchymal cells that are producing extracellular matrix are required for the formation of a functional thymus [43–46]. The mesenchymal cells in the fetal thymus produce soluble factors such as insulin-like growth factor-1 (IGF1), IGF2, fibroblast growth factor-7 (FGF7), FGF10, bone morphogenic protein-4 (BMP4), and the Wnt ligands to facilitate the proliferation and differentiation of TECs [47–52]. This is supported by results that the number of TECs and the size of the thymus significantly decrease when the thymic primordium, from which the outer mesenchymal layer has been removed, is transplanted under the kidney capsule [53]. Mesenchymal cells also exert a negative regulatory effect on TECs, by producing retinoic acid, a vitamin A metabolite that inhibits TEC proliferation [54]. Recently, a series of single-cell RNA-seq analyses of the thymus have been reported, providing comprehensive gene expression profiles of TECs and mesenchymal cells from the embryonic to adult stages [4, 55]. However, the mechanisms by which mesenchymal cells divide into the capsule and medulla and become committed thymic fibroblasts remain unclear and require further analysis at a higher resolution.

### Capsular fibroblast (capFb)

The thymus of the adult mouse is covered with a monolayer of capFbs that contacts the epithelial parenchyma across the basement membrane. capFbs can be distinguished from mFbs by the expression of certain unique genes such as *Dpp4* and *Pi16* in the mouse thymus [56]. On flow cytometry, mouse capFbs are detected as CD45^-^EpCAM^-^CD31^-^PDPN^+^DPP4^+^ cells. The human thymus also contains DPP4-/PI16-expressing capFbs (also termed interlobular fibroblasts), which together with mesothelial cells (also termed perilobular fibroblasts) form the structure of the capsule and the septa that divide the parenchyma into lobes [57]. Although DPP4 is a useful marker to detect capFbs, whether DPP4 is involved in the function of the thymic capsule remains to be elucidated. DPP4 has also recently attracted attention as a marker for activated fibroblasts, and its activity has been reported to be involved in tissue fibrosis [58–62].

capFbs express the Wnt ligand genes at the highest levels among any of the thymic stromal cells [56]. It has been reported that the Wnt signals are required for TEC differentiation, suggesting a role for capFb-derived Wnt signaling in the thymus [51, 52, 63]. In addition, capFbs, together with perithymic mesothelial cells, strongly express genes encoding enzymes for the biogenesis of retinoic acid (*Aldh1a1*, *Aldh1a2*, and *Aldh1a3*), which negatively regulate TEC proliferation and differentiation [64]. It has also been suggested that the semaphorin ligands (*Sema3c* and *Sema3d*) that are highly expressed in capFbs may control thymocyte migration [65]. The outermost structure of the thymus is thought to be organized by capFbs and subcapsular cTECs, but its physiological significance still remains to be clarified, compared with the other stromal cells such as mFbs and mTECs. This is an important issue in the future, including the possibility that capFbs may provide a physical and chemical barrier for the prevention against the entry of cells and antigens from outside into the thymus.

### Medullary fibroblast (mFb)

mFbs form a reticular structure that resembles conduit-like network in secondary lymphoid organs [66]. On histology, the mFb network is tightly interwoven with but separated from the reticular network formed by mTECs. In flow cytometry analysis, they are detected as CD45^-^EpCAM^-^CD31^-^PDPN^+^ DPP4^-^ cells [56]. A fraction of mFbs gives rise to adventitial cells surrounding the blood vessels, forming the perivascular space [67]. Unlike capFbs, isolated mFbs cannot be maintained in a monolayer culture [56]. This may reflect the different nature of these two thymic fibroblast subsets; monolayer capFbs and three-dimensional mFbs.

Studies using lineage-tracing approaches with different Cre lines specific to NC (Sox10-Cre, Twist2-Cre, or Wnt1a-Cre) or TEC (Foxn1-Cre) demonstrated that both capFbs and mFbs are derived from NC-derived mesenchymal cells not from TECs [56, 68, 69]. mFbs are spatially and phenotypically separated from capFbs in the late fetal stages in mice (E14.5) and mature and expand in the postnatal thymus via the interaction with SP thymocytes (as discussed later) [56]. In the thymus of aged mice, the number of mFbs increases [70]. Since it has been suggested that a fraction of the TECs in the aged thymus can trans-differentiate into fibroblasts [71, 72], it may be important to investigate the heterogeneity of mFbs and their possible contribution to the altered microenvironment in the aged thymus.

The maturation of mFbs during the postnatal stages depends on the lymphotoxin signal from SP thymocytes [56]. Lymphotoxin is a heterotrimer ligand composed of two TNF superfamily proteins (LTα and LTβ) and is most highly expressed on SP thymocytes in the thymic medulla, where it binds to the lymphotoxin β receptor (LTβR) to transduce the signals [73–75]. LTβR is expressed at the highest level on mFbs among the thymic stromal cells [56]. Upon the binding to lymphotoxin, LTβR activates the intracellular non-canonical NF-κB pathway. In mice lacking SP thymocytes, LTβR, or NF-κB-inducing kinase, the expression of most mature mFb-specific genes is reduced [56]. The expression of these genes is induced by engaging the LTβR signal in the culture of the fetal thymus. Thus, the LTβR signal plays an essential role in inducing the functional maturation of mFbs.

The LTβR-dependent genes expressed in mFbs include cell adhesion molecules (*Icam1* and *Vcam1*), extracellular matrix proteins (collagens), and chemokines (*Ccl19*), as well as certain TRAs that are not highly expressed in mTECs or other stromal cells in the thymus [56, 67]. Mice specifically lacking the LTβR in fibroblasts show signs of autoimmunity in peripheral tissues, with a marked production of autoantibodies against these mFb-associated TRAs [56]. Indeed, certain TCR clones escape negative selection in fibroblast-specific LTβR-deficient mice. Thus, mature mFbs produce a set of TRAs different from those of mTECs, maximizing the variety of self-antigens in the medulla for the induction of T cell tolerance. mFbs express higher levels of genes for antigen processing and presentation compared with capFbs, suggesting that mFbs contribute to antigen presentation in the thymic medulla [4, 70]. The self-antigens expressed in mFbs may be presented directly by MHC-I to CD8SP thymocytes to induce CD8 T cell tolerance. In addition, it is likely that mFb-specific antigens, including MHC-II-associated antigens, might be transferred to and indirectly presented by thymic dendritic cells to induce CD4 T cell tolerance.

In mice lacking the LTβR in fibroblasts, the number of mTECs is reduced [56]. It was also shown that LTβR-deficient mice exhibit an altered localization of mTECs [76]. Thus, mature mFbs may control the number and localization of mTECs and indirectly promote the induction of T cell tolerance. LTβR signaling in mFbs induces the expression of cell adhesion molecules and proteases that remodel the extracellular matrix, likely contributing to the generation, maintenance, and arrangement of mTECs [56, 67]. The relationship between mFbs and mTECs is unidirectional, because the loss of mTECs does not impair mFbs, either numerically or functionally [56]. However, in mice lacking the LTβR specifically in TECs, the mature mFbs are increased [56], suggesting that mFbs and mTECs compete for the lymphotoxin signaling from SP thymocytes and that the balance between the two stromal lineages is important for the formation of the medullary microenvironment and the induction of self-tolerance. mFbs also control the numbers of mTECs in an LTβR-independent manner via the production of fibroblast-specific protein 1 (FSP1), but its molecular mechanism is not clear [77].

It has also been suggested that mFbs regulate the migration of T-lineage cells through the control of chemokines. Podoplanin (PDPN), a mucin-like glycoprotein highly expressed in mFbs, captures the chemokine CCL21 and displays it on the surface of mFbs, supporting the migration of Treg progenitors to the medulla and their maturation into Tregs [66]. In addition, mFb-derived adventitial cells produce heparan sulfate on their surface that captures CCL21, and the immobilized CCL21 plays a critical role in T cell migration [78]. The cell surface heparan sulfate may also be important for the maintenance of thymic stromal cells, including TECs [79]. mFbs do not produce CCL21, but they highly express other chemokines such as CXCL14 and CX3CL1 [56]. The function of these mFb-associated chemokines in the thymus is not yet known.

### Vascular mural cells

NC-derived mesenchymal cells in the thymus are found as capFbs and mFbs as well as the vascular mural cells surrounding vascular endothelial cells [68, 69]. Endothelial cells are distinguished from other thymic cells by the expression of CD31 (PECAM-1), while mural cells are detected as CD45^-^EpCAM^-^CD31^-^PDPN^-^CD146^+^ cells [4, 80]. Mural cells are classified into vascular smooth muscle cells (VSMCs), which express α-smooth muscle actin (α-SMA) and have contractile activity, and non-contractile pericytes [81, 82]. These cells encapsulate the arterioles and postcapillary venules in the CMJ and medulla (Fig. 1F). In some vessels, endothelial cells and mural cells are surrounded by adventitial cells derived from mFbs. Under reaggregated culture conditions, mFbs have been shown to give rise to mural cells [67], but the intercellular signals and transcriptional mechanisms that control mural cell differentiation remain unidentified.

Since vascular mural cells cannot easily be dissociated from the thymus tissues by collagenase digestion [4], the characterization of these cells at the molecular level has lagged behind that of other thymic stromal cells. Recently, mural cells have been detected by flow cytometry and single-cell transcriptome analysis, and their gene expression signatures have been elucidated [70]. The mural cell population—although it is unclear how heterogeneous it really is—can be distinguished from fibroblasts and endothelial cells by the high expression of muscle-related genes (*Acta2*, *Myl9*, and *Myh11*), regulators of G-protein signaling (*Rgs4*, *Rgs5*, and *Rgs16*), and chemokines (*Ccl19* and *Cxcl1*) [56]. Certain neuron-associated genes (*Nes*, *Pde1a*, and *Pde1b*) are also highly expressed in mural cells in the thymus.

Vascular mural cells, together with adventitial cells, form the perivascular space in which mature T cell egress from the thymus to the periphery. The thymic egress of mature T cells is promoted by the lipid mediator sphingosine-1-phosphate (S1P) that is produced by vascular mural cells and its receptor, the S1P receptor 1 (S1P1) [83]. Mature SP thymocytes in the medulla strongly express S1P1 on the cell surface and are attracted by S1P into the perivascular space. The functional diversity of mural cells is likely related to the diversity of endothelial cells in the thymus. Distinct subsets of thymic endothelial cells associated with the perivascular space have been shown to serve as the entry site for ETPs or exit site for mature T cells [84–86]. Endothelial cells of the capillaries and arterioles in the thymus express claudin-5, tight-junction-forming protein that helps form the blood-thymus-barrier, which insulates the thymic microenvironment from blood-borne molecules, including antigens [87]. However, the complete picture of these vascular cell populations and the molecular mechanisms underlying their diversity remains to be fully elucidated.

## Conclusion

Fibroblasts have generally been considered to be “common” featureless cells that are distributed throughout the organs and tissues of the body. Recently, however, advanced technologies such as single-cell analysis, fate-mapping of differentiating cells, and multi-color cytometry have changed this perception [88–90]. Nowadays, mesenchymal stromal cells, including fibroblasts, are attracting considerable attention as one of the most important “structural cells” in a variety of physiological functions and pathologies due to their remarkable functional diversity [91]. This trend towards a new appreciation also includes the functional characterization of mesenchymal stromal cells in the thymus. The identification of thymic fibroblast subsets and their role in T cell selection has greatly advanced our understanding of the thymic microenvironment. What remains to be elucidated is how thymic fibroblasts interact with other stromal cells, including TECs and mural cells, and how they contribute to not only the formation and maintenance of the thymic microenvironment but also thymic involution and adiposis during stress and/or aging. A better understanding of cellular basis of the entire suite of thymic stromal cell activities will provide key insights into the regeneration of the thymus and thus the functional reconstitution of the immune system.

## Data Availability

Not applicable

## Abbreviations

Aire: Autoimmune regulator
CCL21: C–C motif chemokine ligand 21
CX3CL1: C–X3–C motif chemokine ligand 1
CXCL14: C–X–C motif chemokine ligand 14
DPP4: Dipeptidyl peptidase-4
EpCAM: Epithelial cell adhesion molecule
FoxN1: Forkhead box protein N1
MHC: Major histocompatibility complex
NF-κB: Nuclear factor-kappa B
PECAM-1: Platelet endothelial cell adhesion molecule-1
TNF: Tumor necrosis factor
